# Recycling of Kinesin-1 Motors by Diffusion after Transport

**DOI:** 10.1371/journal.pone.0076081

**Published:** 2013-09-30

**Authors:** T. Lynne Blasius, Nathan Reed, Boris M. Slepchenko, Kristen J. Verhey

**Affiliations:** 1 Department of Cell and Developmental Biology, University of Michigan Medical School, Ann Arbor, Michigan, United States of America; 2 R. D. Berlin Center for Cell Analysis and Modeling, Department of Cell Biology, University of Connecticut Health Center, Farmington, Connecticut, United States of America; CNRS, France

## Abstract

Kinesin motors drive the long-distance anterograde transport of cellular components along microtubule tracks. Kinesin-dependent transport plays a critical role in neurogenesis and neuronal function due to the large distance separating the soma and nerve terminal. The fate of kinesin motors after delivery of their cargoes is unknown but has been postulated to involve degradation at the nerve terminal, recycling via retrograde motors, and/or recycling via diffusion. We set out to test these models concerning the fate of kinesin-1 motors after completion of transport in neuronal cells. We find that kinesin-1 motors are neither degraded nor returned by retrograde motors. By combining mathematical modeling and experimental analysis, we propose a model in which the distribution and recycling of kinesin-1 motors fits a “loose bucket brigade” where individual motors alter between periods of active transport and free diffusion within neuronal processes. These results suggest that individual kinesin-1 motors are utilized for multiple rounds of transport.

## Introduction

Molecular motors that move along microtubule tracks drive the long-distance transport of protein complexes, vesicles, and organelles in cells. In general, kinesin motors drive anterograde transport (towards the cell periphery) whereas cytoplasmic dynein drives retrograde transport (towards the cell center). In recent years, specific cargo molecules for various motors have been identified and mechanisms that regulate motor activity have been described [1]–[5]. Yet the molecular events that occur after completion of transport - release of cargo from motor, retention of cargo at the destination, and the fate of the motor – are poorly understood.

Kinesin-1 (formerly conventional kinesin or KIF5) is a heterotetramer of two subunits, the catalytic kinesin heavy chain (KHC) and accessory kinesin light chain (KLC). When not bound to cargo, kinesin-1 is kept inactive by an autoinhibition mechanism and distributes throughout cells, presumably by simple diffusion. Binding of cargo and/or regulatory proteins to kinesin-1 activates it for motility [3]. One cargo of kinesin-1 in neuronal cells is the JNK-interacting proteins (JIPs), which are scaffolding proteins for the c-Jun N-terminal kinase (JNK) signaling pathway [6]–[10]. The JIPs localize to neurite tips or axonal growth cones and therefore are presumably released from the motor and retained at the destination after completion of transport [7], [11]. Localization of JIP1 in neurite tips is dependent on continuous active transport by kinesin-1 as determined by fluorescence recovery after photobleaching (FRAP) studies [12]. The fate of kinesin-1 after completion of transport has not been studied.

One possibility is that kinesin-1 could be degraded in the axon terminal after completion of transport. This possibility was suggested by studies in which a ligature was applied to bundle of nerves and the accumulation of motors and cargoes on the proximal (closest to the cell body) and distal (closest to the axon terminal) sides of the ligature was determined. While cargoes accumulate on both sides of the ligature, kinesin motors accumulate on the proximal side of the ligature, a finding that has been interpreted as kinesin motors being degraded after transport to the nerve terminal [13]–[24]. However, the relatively long half-life of kinesin motors [25], [26] as compared to the time scale of axonal transport events suggests that motors could be utilized for multiple rounds of transport.

A second possibility is that kinesin motors could be recycled via cytoplasmic dynein-dependent retrograde transport. This possibility is supported by the fact that direct interactions have been identified between kinesin motors and components of the cytoplasmic dynein complex [27], [28] and that kinesin and dynein motors can be co-localized on vesicular cargoes (e.g.[29]–[36]). Although kinesin-dependent transport is required to deliver cytoplasmic dynein to the plus ends of microtubules in the periphery [37]–[42], the fact that kinesin motors do not accumulate on the distal side of a ligature argues against kinesins being carried as “transport cargos” of motors moving back to the cell center [13]–[24]. Thus, the co-localization and interactions of anterograde and retrograde motors may play a role only in the bi-directional transport of membranous cargoes [43], [44].

A third possibility is that kinesin motors could be recycled by diffusion back to the site of cargo loading. A significant portion of both cytoplasmic dynein and kinesin motors have been found in soluble cytosolic extracts and these motors likely localize throughout cells by diffusion (e.g [28], [30], [45]–[49]). Diffusion of soluble motors seems likely to facilitate kinesin recycling in spherical fibroblast cells but may not be sufficient for motor distribution in spatially restricted compartments such as axons and cilia.

We set out to test these models concerning the fate of kinesin-1 motors after completion of transport in neuronal cells. We find that kinesin-1 motors are neither degraded nor returned by retrograde motors. Rather, we show that the distribution and recycling of kinesin-1 motors fits a “loose bucket brigade” where the term bucket brigade refers to the passing of items from one worker to another in sequence. For kinesin-1 motors, the term loose bucket brigade implies that motors do not necessarily act in sequence but that individual motors participate in periods of active transport interspersed with periods of free diffusion within neuronal processes.

## Results

### Kinesin-1 is not rapidly degraded after transport

**Figure 1:**
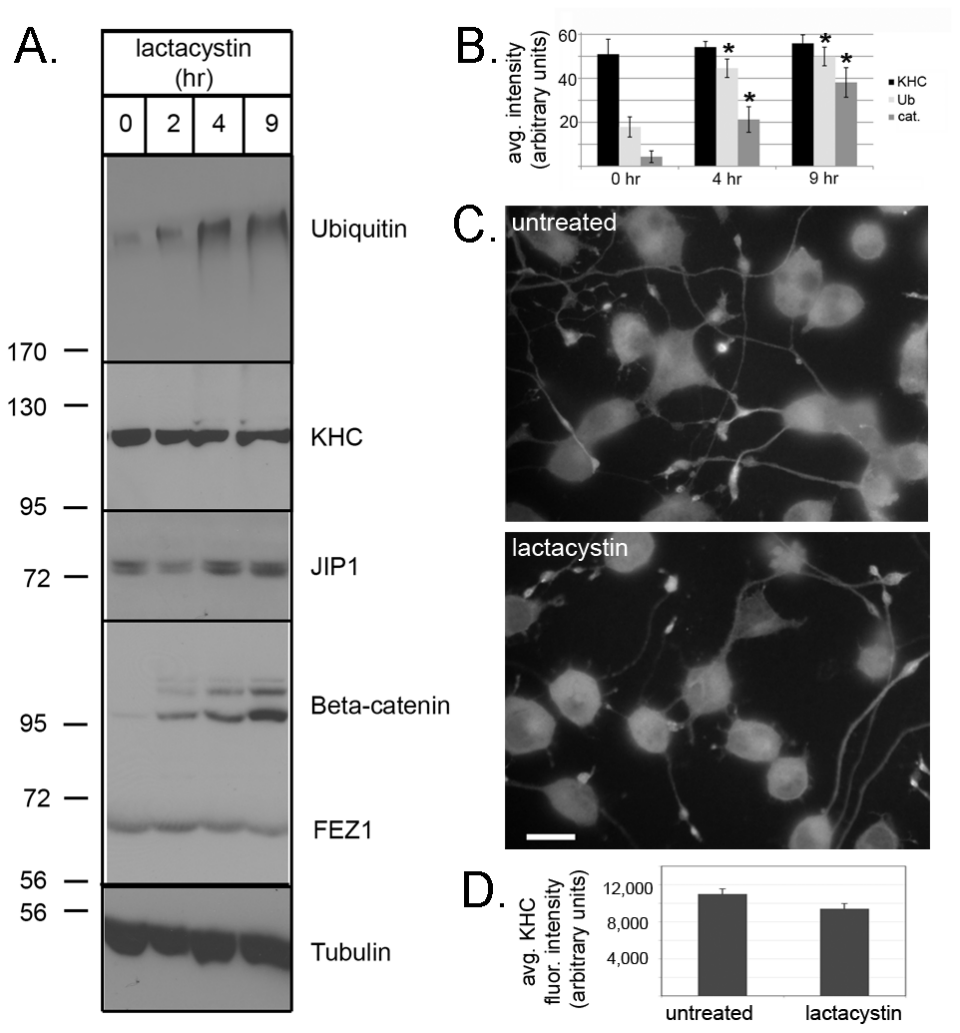
Kinesin-1 is not rapidly degraded by the proteasome. (A,B) Differentiated CAD cells were treated with 5 µM lactacystin for the indicated times. (A) Soluble protein lysates were immunoblotted to detect total levels of ubiquitinated proteins (ubiquitin), KHC, JIP1, β-catenin, FEZ1, and tubulin. (B) The average levels of KHC (black bars), ubiquitin (light gray bars) and β-catenin (dark gray bars) were quantified from 6–8 experiments. Error bars indicate SEM. *, p<0.01 as compared to 0 hr. (C,D) Differentiated CAD cells were untreated (top panel) or were treated with 5 µM lactacystin for 6 hrs (bottom panel) then fixed and stained with a monoclonal antibody to endogenous KHC. Scale bar, 15 µm. The average fluorescence intensity of endogenous KHC at neurite tips was quantified (D) in untreated and lactacystin-treated cells. *n* = 15 cells from two independent experiments for each condition. Error bars indicate SEM. p = 0.055 for treated versus untreated cells.

We first set out to test if kinesin-1 is degraded after completion of transport. We reasoned that if this possibility is correct, then pharmacological treatments that block protein degradation should result in an increase in kinesin-1 protein levels. Differentiated neuronal CAD cells or primary hippocampal neurons were treated with the proteasome inhibitors lactacystin or MG132 and total protein levels in cell lysates were analyzed by western blotting. As expected, lactacystin (Figure 1 A,B) or MG132 (Figure S1 A,B) treatment resulted in an increase in the total amount of ubiquitinated proteins as well as a specific proteasome substrate, β-catenin [50]. In contrast, proteasome inhibition had no effect on the total level of the KHC subunit of kinesin-1 nor on the total amounts of the kinesin-1-associated proteins JIP1 and fasciculation and elongation PKCζ-interacting protein 1 (FEZ1; [51]).

To test whether proteasome inhibition could alter the subcellular localization of motors that have completed transport despite no change in overall protein levels, we performed immunostaining of untreated and treated CAD cells. In untreated cells, the KHC subunit was localized diffusely throughout cells and accumulated at the tips of most neurites. Treatment of cells with the proteasome inhibitors lactacystin (Figure 1 C,D) or MG132 (data not shown) caused no change in the localization of KHC. The amount and localization of kinesin-1 motors were also unaffected by treatment of differentiated CAD cells with inhibitors of calpain proteases (Figure S1 C–E). Taken together, these results suggest that kinesin-1 is not degraded after completion of transport in cultured neuronal cells.

### Retrograde motors do not play a major role in return of kinesin-1 motors

We next tested the second possibility as to the fate of kinesin motors - that kinesin-1 is recycled for further rounds of transport via retrograde motors. We reasoned that if cytoplasmic dynein function is required for returning kinesin-1 motors to the cell body, then disruption of cytoplasmic dynein function would result in an accumulation of kinesin-1 motors at neurite tips. In initial experiments, we carried out disruption of cytoplasmic dynein function by overexpression of various dominant negative (DN) constructs in differentiated CAD cells. Overexpression of p50/dynamitin or the N-terminal 237 amino acids (N237) of the dynein intermediate chain 2 (IC2) resulted in dispersion of the Golgi complex (Figure S2A and data not shown), verifying that cytoplasmic dynein function was disrupted [52]–[55]. However, we did not observe an accumulation in kinesin-1 protein at neurite tips upon expression of dynein DN constructs. Rather, kinesin-1 localization was unchanged (Figure S2 B,D and data not shown). Expression of DN forms of minus end-directed kinesin-14 motors also did not result in an accumulation of kinesin-1 motors at neurite tips (Figure S2 C,D). These results suggest that long-term inhibition of retrograde motors does not alter kinesin-1 localization.

**Figure 2:**
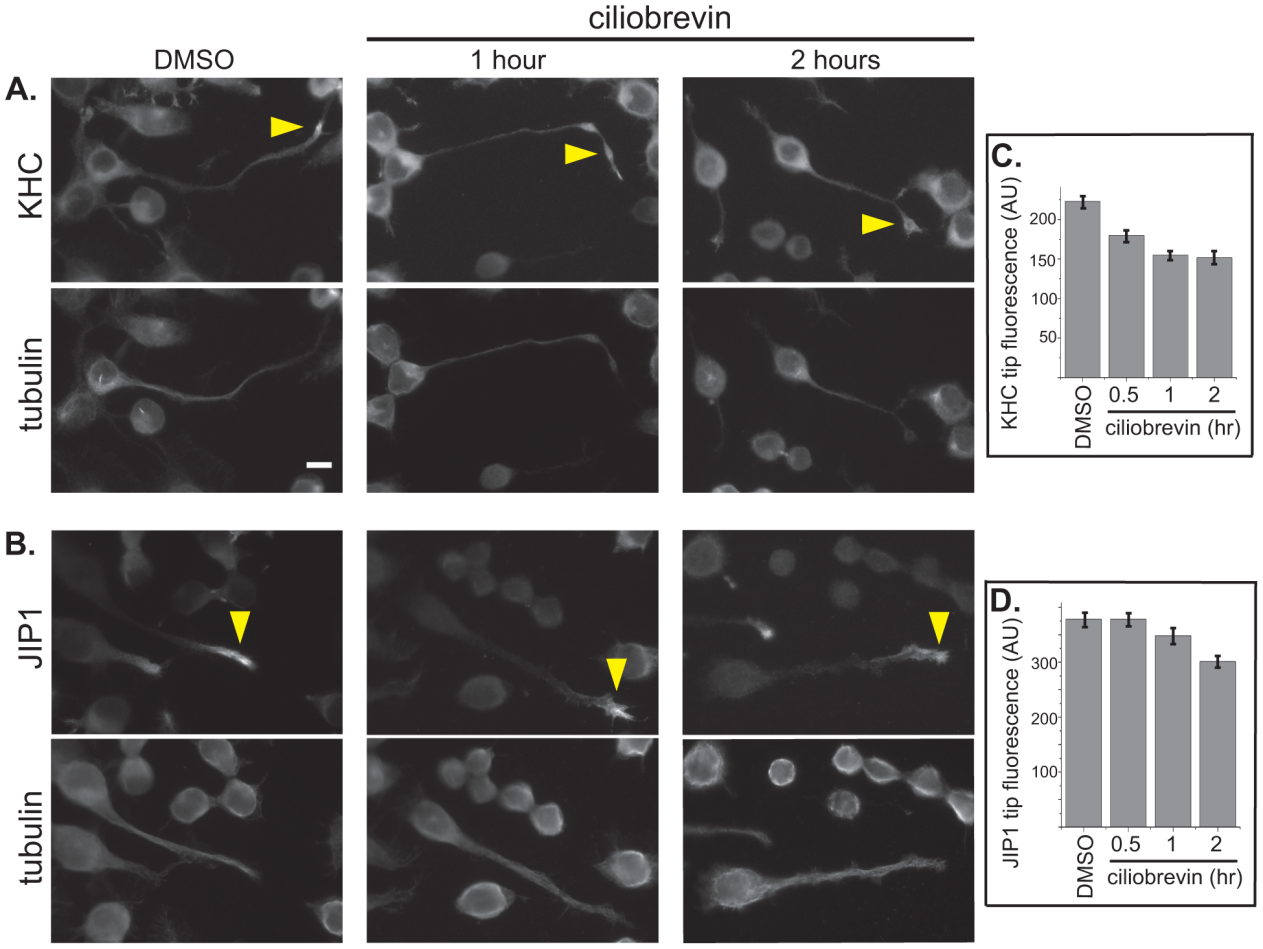
Kinesin-1 recycling does not depend on cytoplasmic dynein. (**A,B**) Differentiated CAD cells were treated with DMSO control for 2 h or with the cytoplasmic dynein inhibitor ciliobrevin A for 0.5 h, 1 h, or 2 h. The cells were then fixed and co-stained with (A) antibodies to the KHC subunit of kinesin-1 and tubulin or (B) antibodies to the kinesin-1 cargo protein JIP1 and tubulin. Yellow arrowheads, KHC or JIP1 at neurite tips. Scale bar, 10 µm. (C,D) Quantification of the average fluorescence intensity of (C) KHC or (D) JIP1 in neurite tips. AU, arbitrary units. For (C), n = 194 cells (DMSO), 186 cells (0.5 h), 158 cells (1 h), or 109 cells (2 h) over two to three independent experiments. For (D), n = 91 cells (DMSO), 84 cells (0.5 h), 95 cells (1 h), or 84 cells (2 h) over three independent experiments. Error bars indicate SEM.

To examine whether cytoplasmic dynein plays a role in recycling of kinesin-1 motors on a short time scale, we treated differentiated CAD cells with ciliobrevin A, a small molecule inhibitor of the catalytic heavy chain subunit of cytoplasmic dynein [56]. Again, inhibition of cytoplasmic dynein function did not cause kinesin-1 motors to accumulate in neurite tips (Figure 2 A,C). These results indicate that cytoplasmic dynein cannot be solely involved in retrograde transport of kinesin-1. Rather, short-term inhibition of dynein function resulted in a decrease in the amount of the kinesin-1 subunit KHC at neurite tips (Figure 2 A,C). Identical results were obtained upon treatment of differentiated CAD cells with ciliobrevin D (data not shown). We wondered whether inhibition of cytoplasmic dynein function caused a concomitant block in anterograde transport, as has been suggested in the literature [57]–[62], as this could explain the decrease in kinesin-1 levels in neurite tips upon ciliobrevin treatment. Indeed, ciliobrevin A treatment caused a block in kinesin-1 transport as the amount of the JIP1 cargo protein at neurite tips also decreased (Figure 2 B,D). The difference in the rate of loss of kinesin-1 versus JIP1 likely reflects the different lifetimes of the motor and its cargo at neurite tips. The fact that long-term inhibition of dynein function has no effect on kinesin-1 localization whereas short-term inhibition results in a decrease in kinesin-1 at neurite tips likely reflects cellular adaptation to loss of the retrograde motor in the long-term situation. Taken together, the results on cytoplasmic dynein inhibition indicate that this motor is not required for retrograde transport of kinesin-1 motors after delivery of cargoes to neurite tips.

### Kinesin-1 motors return to the cell body

As kinesin-1 motors appear to be neither rapidly degraded (Figures 1 and S1) nor transported back to the cell body by retrograde motors (Figures 2 and S2), we next considered the third possibility as to the fate of kinesin motors after transport – that kinesin-1 returns to the cell body by diffusion. To directly test whether kinesin-1 motors that have undergone transport to neurite tips can return to the cell body, the KHC subunit was tagged with photoactivatable green fluorescent protein (PAGFP) and expressed in differentiated CAD cells. Control experiments demonstrated that the FP tag did not disturb the localization, function or interactions of KHC (data not shown), consistent with previous work [51], [63]. Only cells expressing low levels of FP-tagged motors were analyzed (as in Figure S3) to ensure that the dynamics of the PAGFP-tagged subunit reflects movement of endogenous kinesin-1 molecules rather than the expressed KHC subunits.

**Figure 3:**
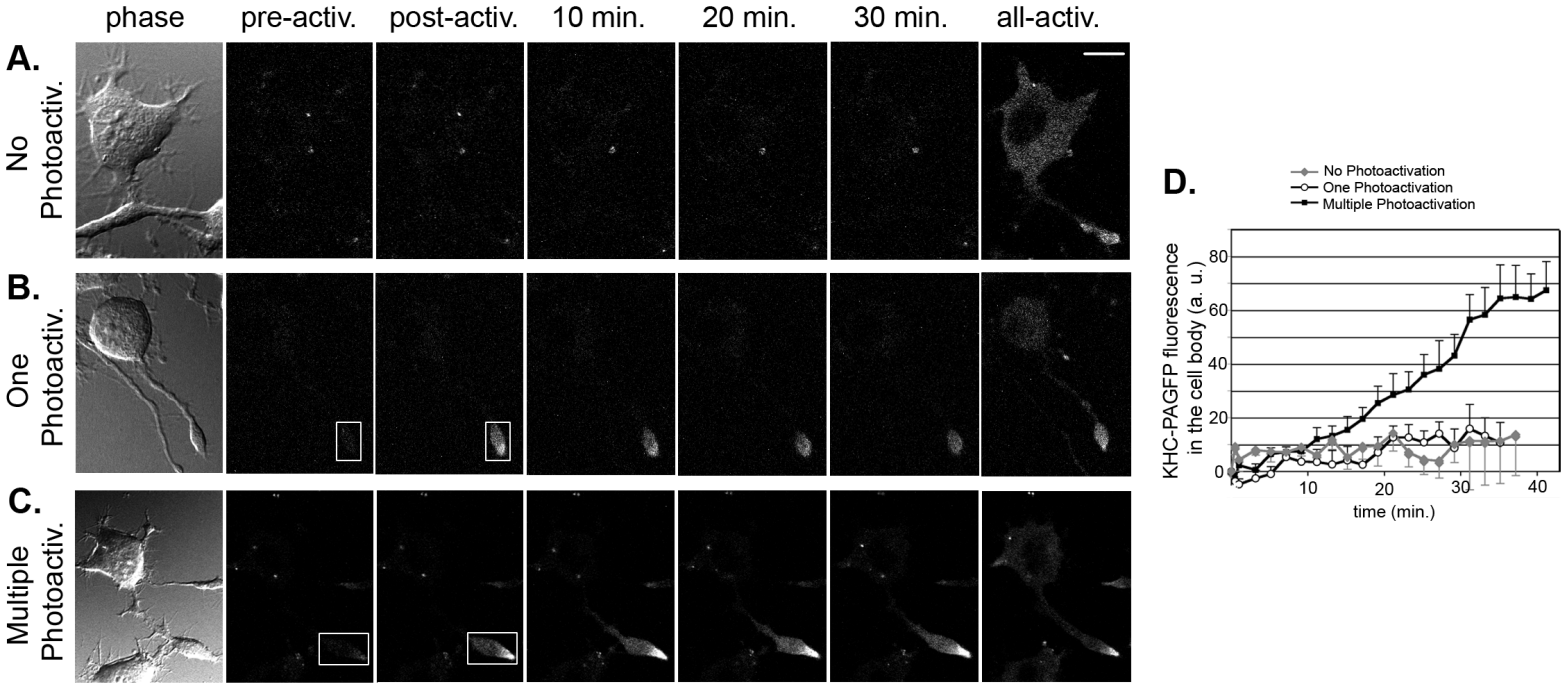
Kinesin-1 returns to the cell body from neurite tips. Differentiated CAD cells were transfected with a plasmid encoding PAGFP-tagged KHC. 48 hr later, the cells were imaged by confocal microscopy. Representative times series from three different imaging conditions are shown. (A) No photoactivation. GFP images of the entire field of view were collected over time. In the final image, the total level of expressed PAGFP-KHC was determined by photoactivation of the entire field of view (all-activ.). (B) One photoactivation event. GFP images were collected (pre-activ.) and then PAGFP-KHC in one neurite tip (white box) was photoactivated (post-activ.) followed by imaging of GFP fluorescence for the entire field of view over time. In the final image, the total level of expressed PAGFP-KHC was determined by photoactivation of the entire field of view (all-activ.). (C) Multiple photoactivation events. GFP images were collected (pre-activ.) and then PAGFP-KHC in one neurite tip (white box) was photoactivated (post-activ.) followed by imaging of GFP fluorescence for the entire field of view for 4 min. The neurite tip photoactivation and whole field GFP imaging was then repeated 9 times. In the final image, the total level of expressed PAGFP-KHC was determined by photoactivation of the entire field of view (all-activ.). Scale bar, 10 µm. (D) Quantification of the average PAGFP-KHC fluorescence in the cell body over time. At least 7 cells in two to three independent experiments were imaged for each condition. Error bars indicate SEM.

To determine whether kinesin-1 motors can return to the cell body, KHC-PAGFP motors in the neurite tip were photoactivated with a 405 nm laser and the GFP fluorescence in the cell body was monitored over time. At the end of the imaging series, the entire field of view was photoactivated (Figure 3, all-activ. panel) to verify that KHC-PAGFP was expressed at low levels and with a distribution similar to that of the endogenous kinesin-1 protein. Cells expressing KHC-PAGFP and imaged without photoactivation showed no change in GFP fluorescence in the cell body (Figure 3 A,D). Thus, the imaging parameters themselves do not cause increased fluorescence over time. When cells expressing KHC-PAGFP were photoactivated one time at the start of imaging, little to no increase in KHC-PAGFP fluorescence in the cell body was detected (Figure 3 B,D). However, when the neurite tip was repeatedly photoactivated during the imaging sequence, an increase of KHC-PAGFP fluorescence in the cell body was observed (Figure 3 C,D). These results suggest that kinesin-1 motors in neurite tips can return to the cell body.

### Kinesin-1 diffusion is similar to that of an inert molecule

To explore the possibility that kinesin-1 motors return to the cell body by diffusion, we first determined the diffusion coefficient for FP-tagged kinesin-1 motors in CAD cell neurites. Unlike the three dimensional (3D) space of fibroblast-like cells and the neuronal cell body, the diffusion of kinesin-1 in the axonal process of a neuron is essentially one dimensional (1D) diffusion due to the extended length of the process as compared to the depth and height. The KHC subunit of kinesin-1 was tagged with monomeric Citrine (mCit), a variant of yellow FP [63], at the N- or C-terminus (mCit-KHC and KHC-mCit, respectively). Similar results were obtained with both tagged KHC motors (data not shown). To generate a control protein that moves only by diffusion (i.e. does not interact with microtubules or cargoes) and has approximately the same molecular weight as kinesin-1, we fused mCit to glutathione S-transferase and Nus (mCit-GST-NUS), two bacterial proteins that are inert in mammalian cells.

**Figure 4:**
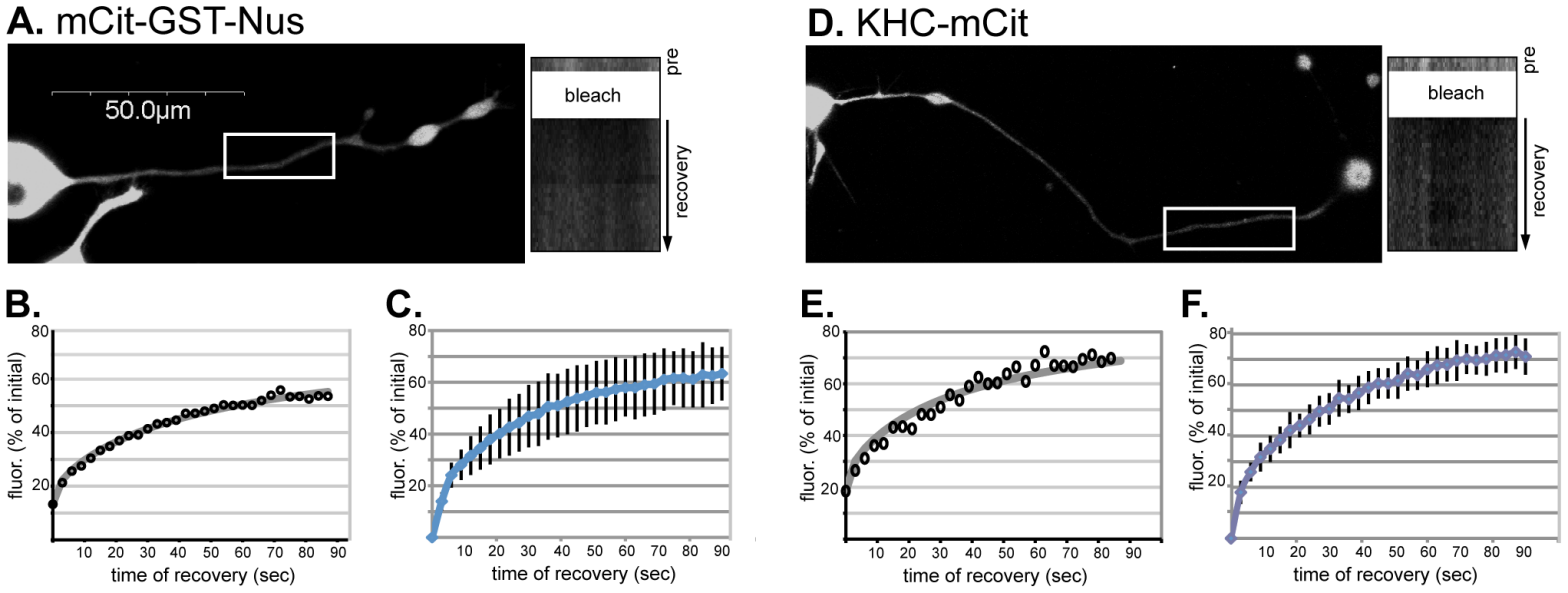
Diffusion of KHC-mCit in neurites. Differentiated CAD cells expressing (A–C) the control mCit-GST-NUS fusion protein or (D–F) mCit-KHC were imaged by confocal microscopy. Low-expressing cells were chosen for imaging but the brightness of the image was digitally enhanced to aid in visualization. A 30–40 µm stretch in the middle of the neurite (white boxed region) was photobleached and then the fluorescence recovery was monitored over time. (A,D) Representative pre-bleach images. Kymographs of fluorescence recovery in the bleached region (right panels) were generated by drawing a line along the bleached region of the neurite (white box) and plotting the line from each time point down the y-axis. (B,E) Graphs of fluorescence recovery in the bleached regions of the representative cells in (A,D). The fluorescence recovery data (black circles) were fit by an analytical solution (gray lines). (C,F) The average fluorescence recovery for multiple cells expressing (C) mCit-GST-Nus or (F) mCit-KHC. *n* = 8 cells each.

Differentiated CAD cells expressing mCit-KHC or mCit-GST-NUS were imaged by confocal microscopy and the diffusion rates were calculated by measuring the fluorescence recovery after photobleaching (FRAP) in the neurites. Only cells expressing low levels of FP-tagged motors were analyzed (Figure S3) so that the measurement of mCit-KHC reflects movement of endogenous kinesin-1 complexes rather than expressed KHC subunits. Three pre-bleach images were obtained, a 30–40 µm long segment in the middle of a neurite was bleached at high laser power, and then the recovery of fluorescence in the bleached area was measured over time. For both mCit-GST-NUS and mCit-KHC, rapid fluorescence recovery was observed (Figure 4), suggesting that soluble kinesin-1 motors move by free diffusion in the neurites of differentiated CAD cells.

To determine the diffusion coefficient of kinesin-1, the fluorescence recovery data for each cell were fit by an analytical solution over the entire measurement time (Figure S4). Averaging the data from 8 cells over the 30 sec recovery period (Figure 4F) resulted in an estimate of 6.4±0.9 µm^2^/s (avg ± std. dev) for the kinesin-1 diffusion coefficient. An average error of fit (the mean absolute difference between the data and the fitting curve), was relatively large (0.035), likely indicating overestimation of the diffusion coefficient due to a contribution from active transport to the fluorescence recovery.

To minimize the contribution from directed transport, we took into account that on short time and distance scales, diffusion is faster than directed transport. We therefore applied the same fitting procedure to the data for the first 15 seconds of the fluorescence recovery period (Figure S4). In this case, the estimated diffusion constant for kinesin-1 was 4.08±0.46 µm^2^/s with the average absolute error of fit of 0.016. This number is close to 4.11±0.54 µm^2^/s, an estimate for the diffusion coefficient of mCit-GST-NUS obtained by applying the same fitting procedure to the recovery data over the entire measurement time. We therefore believe that 4.08±0.46 µm^2^/s is an accurate estimate of the diffusion coefficient of kinesin-1 in the one-dimensional neurite of differentiated CAD cells.

### Modeling of kinesin-1 distribution and transport suggests a Loose Bucket Brigade

To address whether diffusion is sufficient to recycle kinesin-1 motors for further rounds of transport, we used mathematical modeling of kinesin-1 transport events in neuronal processes. All of the models are based on the assumption that when bound to cargo, kinesin-1 undergoes active transport towards the microtubule plus ends with a velocity v = 0.78 µm/sec whereas soluble motors are inactive [63]–[65] and undergo free diffusion at the experimentally-determined diffusion constant of D = 4.08±0.46 µm^2^/s. The equations and assumptions of the models are described in Figure S5, part I.

**Figure 5:**
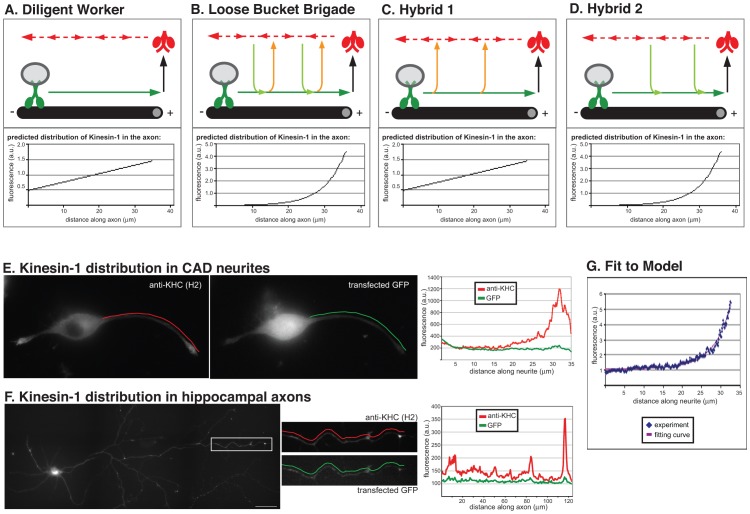
The distribution of kinesin-1 fits with a Loose Bucket Brigade. (A–D) Models for kinesin-1 transport and recycling in neuronal processes. In the Diligent Worker model (A), active kinesin-1 motors (green) transport vesicular cargoes to the plus ends of the microtubules (solid green arrow). The motors disengage from the cargo and track (black arrow), assume the inactive autoinhibited conformation (red), and diffuse in the neurite (dotted red arrows). In the Loose Bucket Brigade (B), active kinesin-1 motors (green) transport vesicular cargoes to the plus ends of the microtubules (solid green arrow) but stochastically fall off the cargo (orange arrows). These motors diffuse within the neurite (dotted red arrows) and can reattach to cargoes (green arrows) and again contribute to the transport of vesicles to the plus ends of microtubules. The Hybrid 1 model (C) assumes that kinesin-1 motors can stochastically fall off the cargo (orange arrows) but cannot reattach while diffusing in the neurite (like the Sloppy Bucket Brigade during active transport and the Diligent Worker during recycling). The Hybrid 2 model (D) assumes that kinesin-1 motors do not fall off during transport but can reattach to cargoes while diffusing in the neurite (like the Diligent Worker during active transport and the Sloppy Bucket Brigade during recycling). For each model, computer simulations were used to determine the predicted distribution of kinesin-1 motors in neuronal processes (bottom panels). (E, F) Experimental determination of kinesin-1 distribution in neuronal processes. (E) Differentiated CAD cells or (F) primary hippocampal neurons expressing EGFP (a marker of cell volume) were stained with an antibody to KHC. The fluorescence intensity of KHC (red lines) and EGFP (green lines) was plotted along the length of the neurite or axon (right panels). The fluorescence intensity of KHC represents the spatial distribution of the total kinesin concentration (a sum of concentrations of bound and freely diffusing kinesin-1). (G) The increasing portion of the kinesin-1 profile (red line) in the neuronal process of the CAD cell in (E) was plotted point-by-point as a ratio of the local fluorescence intensities of KHC and EGFP. The distribution fits with an exponential curve (blue line) and is consistent with the Loose Bucket Brigade model.

In the simplest model, kinesin-1 motors exist in either of two populations, bound to cargo and undergoing active transport all the way to the plus ends of microtubules in the axon tip or unbound from both cargo and microtubule and returning to the cell body by diffusion (Figure 5A, the Diligent Worker model). Mathematical modeling shows that Diligent Workers are effective at transporting cargoes to the axon tips and for non-neuronal cells and/or short processes (<∼10 µm), diffusion does not limit their recycling (Figure S5, Part 1A). Even for longer processes (60–70 µm), recycling of kinesin-1 motors would take ∼10 min, a time frame compatible with that of kinesin-1 transport events [12]. However, in the Diligent Worker model, the recycling of kinesin-1 motors by diffusion is limited in cells with longer processes.

We thus considered a second model where anterograde and retrograde kinesin-1 motors participate in both active transport and diffusion (Figure 5B). In this model, motors bind cargo in the cell body and undergo active transport towards the microtubule plus ends but can stochastically fall off of the cargo and diffuse within the neurite before rebinding to cargo and again contributing to transport. Thus, this model resembles a “bucket brigade” as the cargo frequently “changes hands” between driving motors. We consider this model a “loose” bucket brigade since each motor can diffuse within the axon in both directions and is thus not restricted to working only in a specific segment as would occur in an “organized” bucket brigade. The Loose Bucket Brigade is mathematically similar to models used earlier to describe kinesin distributions in *in vitro* microtubule assays [66] and transport of cargoes in cells [67]. Mathematical modeling of the Loose Bucket Brigade (Figure S5, Part 1B) indicates that diffusion is sufficient for recycling of kinesin-1 motors as the time per kinesin-1 transport cycle is independent of axon length.

To test the Diligent Worker and Loose Bucket Brigade models of kinesin-1 transport, we used the equations in Figure S5 to predict the distribution of kinesin-1 motors in axon and then compared these results to experimentally-determined values. We also considered two hybrid models. In the third model (Figure 5C, Hybrid 1), kinesin-1 motors undergo periods of active anterograde transport interspersed with periods of diffusion (the Loose Bucket Brigade) but upon arrival at the end of an axon, they assume an inactive state that diffuses back to the cell body without participating in additional transport events. In the fourth model (Figure 5D, Hybrid 2), cargo-bound kinesin-1 motors undergo active transport to the neurite tip (the Diligent Worker) but can be recruited to cargoes and participate in further rounds of transport during diffusion back to the cell body. All four models predict higher levels of kinesin-1 motors in the distal region of the axon (Figure 5, A–D). However, both the Diligent Worker and the Hybrid 1 models predict a linear increase in total kinesin-1 density along the axon whereas the Loose Bucket Brigade and Hybrid 2 models predict an exponentially increasing density of kinesin-1 along the axon.

To experimentally determine the distribution of kinesin-1 molecules, neuronal cells were immunostained using a monoclonal antibody (H2) that recognizes all three KHC gene products (KIF5A, KIF5B and KIF5C) [68]. The distribution of kinesin-1 was determined in differentiated CAD cells which generate neurite processes and in primary hippocampal neurons which generate true axons and dendrites (Figure S5, Part 2). To correct for fluorescence variation due to changes in cell volume, the cells were first transfected with plasmids to express EGFP and then fixed and stained for kinesin-1. The typical qualitative feature of the KHC distribution is the increase in total kinesin-1 concentration at the tip of the process in both CAD cells and primary hippocampal neurons (Figure 5 E,F). Quantification of the fluorescence intensity from the base of the cell body to the tip of the neurite for the CAD cell in Figure 5E shows an exponential increase in kinesin-1 protein levels (Figure 5G and Figure S5B). A similar exponential distribution was found across 12 CAD cell neurites and 18 hippocampal axons (Figure S5 and data not shown). This finding decisively discriminates between the models of kinesin-1 distribution. That is, the characteristic exponential increase in kinesin-1 distribution at the neurite tip can be explained only by the Loose Bucket Brigade and the Hybrid 2 models. The distinguishing feature between these two models, whether kinesin-1 motors come off of cargoes during their transit down the axon, is currently unknown. When fit to the experimental data (Figure 5G), the Loose Bucket Brigade model yields the rate of binding to the microtubule 1.22 s^−1^ (Figure S5), based on the previously measured rate of motor inactivation 0.67 s^−1^ and velocity 0.78 µm/s [65] and the diffusion coefficient 4.08 µm^2^/s from the preceding section.

## Discussion

Molecular motors actively transport many types of cargo along cytoskeletal filaments in a wide range of organisms. Upon delivery of a cargo to its destination, the fate of the motor is poorly understood. Our results suggest that kinesin-1 motors can be recycled for further rounds of transport by diffusion of inactive motors in the axon.

We propose a model in which kinesin-1 transport in neuronal cell processes occurs by a Loose Bucket Brigade (Figure 5B). In this model, individual kinesin-1 motors detach from the cargo stochastically during transport in the axon and upon reaching the destination. Detached motors resume a folded inactive conformation and undergo free diffusion in the axon where they can stochastically reattach to cargoes and again contribute to active transport. While only a small fraction of kinesin-1 motors return all the way to the cell body, consistent with our live cell imaging in Figure 3, individual kinesin-1 motors are recycled by diffusion and participate in multiple rounds of transport.

Bucket brigades are best known for their usage at the population level. Bucket brigades are highly efficient at coordinating order-pickers in a distribution center [69] and the collection of resources in insect colonies [70]. Bucket brigades have been noted previously at the molecular scale in the transfer of copper to the COX holoenzyme and the movement of iron to ferritin catalytic sites [71], [72]. For axonal transport, one particularly attractive aspect of a bucket brigade is that it is self-organizing and thus results in a dynamically ordered system from the interactions of the individual parts.

### Recycling of kinesin-1 motors by diffusion

Our results rule out the possibility that kinesin-1 motors are degraded at the nerve terminal. This is consistent with the time frame of kinesin protein turnover versus that of kinesin-dependent transport events. Kinesin motors appear to be long-lived proteins (half-life >20 h, [25], [26]) whereas kinesin-dependent axonal transport events occur on the time scale of hours or less ([12], [64], data not shown). It thus appears that individual kinesin-1 motors participate in multiple rounds of transport.

Our results also rule out the possibility that kinesin-1 motors return to the cell body by dynein-dependent retrograde transport. We cannot exclude the possibility that some kinesin-1 motors can be carried as cargo by dynein or kinesin-14 motors while the bulk of the kinesin-1 motors undergo free diffusion in the cytoplasm. Kinesin-1 has been suggested to interact with dynein, both directly and indirectly, yet the functional roles of these interactions are still unclear [27], [28], [73]. We suggest that the co-localization of kinesin-1 and dynein motors on the same cargo is required for rapid changes in cargo transport direction [43], [44] but not for recycling of kinesin motors from the nerve terminal.

That kinesin-1 motors are neither degraded upon reaching the nerve terminal nor returned to the cell body by minus end-directed motors is consistent with the results of nerve ligature experiments. Both retrograde motors and cargoes have been found to accumulate on the distal side of the ligature, consistent with their undergoing active transport back to the cell body. In contrast, kinesin motors largely accumulate at the proximal side of the ligature, indicating that active transport in the retrograde direction is not the major route of kinesin recycling [13]–[24].

We provide direct evidence for the possibility that kinesin-1 motors return to the cell body after transport. In addition, we show that, on a short time scale, FP-tagged kinesin-1 motors undergo free diffusion in neuronal cell processes at a rate consistent with that of similarly-sized proteins and dextrans [74]. Finally, our modeling suggests that the recycling of kinesin-1 motors by diffusion for multiple rounds of transport is consistent with the experimentally-observed exponential increase in kinesin-1 protein at axon terminals as a characteristic of the loose bucket brigade mechanism.

### Implications of the Loose Bucket Brigade for kinesin-1 transport

One implication of the loose bucket brigade is that kinesin-1 motors interact transiently with their cargoes during transport down an axon. Any modeling that incorporated a stable interaction of motors with cargoes from the cell body to the axon terminal (i.e. Diligent Workers) was unable to replicate the exponential increase in endogenous kinesin-1 motors at the nerve terminal. At first glance, a transient interaction of kinesin-1 motors with cargoes appears incompatible with the fact that a complement of motors remains tightly associated with membranes throughout purification (see e.g. [30], [37], [45], [46], [75]–[81]). However, depending on buffer conditions, up to 70% of cellular kinesin-1 remains in the soluble fraction [28], [30], [45]–[48] and the interchange of motors between the tightly bound and cytoplasmic pools in cells is unknown. That is, to our knowledge, whether kinesin-1 motors interact transiently with their cargoes during transport has not been directly tested. Recent work suggests that kinesin-3 and dynein do interact transiently with their cargoes in fungi as live cell imaging demonstrated that fluorescently-tagged motors come on and off their cargoes during anterograde and retrograde transport events [82].

A second implication of the loose bucket brigade is that only a fraction of kinesin-1 motors are bound to cargo at any time (Figure S5, part 1). This is compatible with studies indicating that only a small number of kinesin-1 motors are associated with individual cargoes [29]–[36], [83]. Tagged kinesin-1 constructs expressed exogenously in neuronal and other cells displays a largely cytoplasmic localization with little to no labeling of vesicles or organelles [84], [85]. However, the amount of kinesin-1 protein associated with membrane cargoes versus free in the cytoplasm has been controversial. Depending on the buffer conditions used for solubilization or the antibody used for staining, kinesin-1 can appear mostly cytoplasmic or mostly membrane-bound [28]–[30], [45]–[48]. Our modeling indicates that the observed localization gradient of kinesin-1 in neuronal cells is defined, among other factors, by the rates of kinesin-1 association with and dissociation from cargoes. Thus, understanding how kinesin-1 binds to and detaches from its cargoes during transport events is an important open question.

A third implication of the Loose Bucket Brigade is that the return of kinesin-1 motors to the cell body is limited and thus, the targeting of cargoes in the cell body appears ineffective. This is most likely due to the fact that, even in the Loose Bucket Brigade, diffusional recycling is not effective over long distances [74], [86]–[88]. Several mechanisms can be envisioned to overcome the limits of diffusion in long neuronal processes. First, cells with long neuronal processes may up regulate a dynein-dependent return of kinesin motors that is not evident in our cultured cells. Second, neuronal cells with long axons may have higher levels of kinesin-1 synthesis, both in the cell body and within axonal processes, to meet the cell's axonal transport needs. In support of this, kinesin-1 mRNA has been found in axons [89]. Third, it may be necessary to restrict the free diffusion of kinesin-1 within the axon. That is, the brigade may be ‘organized’ through ‘diffusion rectification’ such that individual motors are not allowed to diffuse in the “wrong” direction by some kind of diffusion barriers – these could be pile-ups of large cargoes either due to disruptions of microtubule tracks or due to stochastic cargo jams [90]. In the latter case, the diffusion barriers could dissipate and reappear in different places.

### The fate of other kinesin motors after transport

In addition to kinesin-1, members of several other kinesin families participate in axonal transport events [91]. For the kinesin-2 family, several lines of evidence suggest that these motors are also not recycled by cytoplasmic dynein in neuronal cells. First, nerve ligature experiments have demonstrated that kinesin-2 motors do not accumulate on the distal side of the ligature [20]–[22]. Second, the recycling of fluorescently-tagged kinesin-2 motors in the dendrites of *C. elegans* chemosensory neurons was not altered by loss-of-function mutation in one of the two cytoplasmic dynein heavy chain subunits [92].

Kinesin-3 family motors carry a variety of transport cargoes in neurons, such as synaptic vesicles and dense core vesicles [93], yet their fates upon arrival at the axon terminal are unclear. Studies in worms, fungi and cultured mammalian neurons have shown that fluorescently-tagged kinesin-3 motors are present on puncta moving in both the anterograde and retrograde directions [32], [37], [94]–[96]. Whether these observations indicate motors involved in the bidirectional movement of cargoes or motors that undergo transport in the opposite direction is unclear. Nerve ligature experiments indicate that kinesin-3 motors are not recycled by cytoplasmic dynein-dependent transport as they do not accumulate on the distal side of the ligature [18]. In addition, a recent study in *C. elegans* suggested that the kinesin-3 motor UNC-104 is not retrogradely transported by cytoplasmic dynein [97]. However, these results appear to contradict previous work from the same group showing that mutations in various cytoplasmic dynein subunits caused an accumulation of UNC-104 at the ends of neuronal processes [98]. Whether kinesin-3 motors are degraded after transport is also controversial and data exists arguing for and against a role for proteosome-mediated degradation [37], [97]. Clearly, more work is required to understand the fate of kinesin motors after the completion of transport.

## Materials and Methods

### Cell Culture and Epifluorescence Microscopy

The CNS catecholaminergic cell line CAD (Cath.a-differentiated), a gift of Dr Dona Chikaraishi [99], was cultured, differentiated and transfected as described previously [12] except that TransIT-LT1 (Mirus) was used for transfection. Primary hippocampal neuron (PHN) cultures were prepared from embryonic day 16 CD1 mice as described previously [100], [101]. Transfection of DNA plasmids was done at the time of plating using a nucleofection protocol (Amaxa Biosystems, Gaithersburg, MD). All mouse work was carried out in strict accordance with the recommendations in the Guide for the Care and Use of Laboratory Animals of the National Institutes of Health. The protocol was approved by the University of Michigan Institutional Animal Care and Use Committee (IACUC) (number 09724). All efforts were made to minimize animal stress and suffering.

Cells were fixed in 3.7% paraformaldehyde at room temperature or 100% methanol at −20°C, permeabilized in 0.2% Triton X-100 in PBS, blocked in 0.2% fish skin gelatin in PBS, stained, and mounted in ProLong Gold (Invitrogen). Images of fixed cells were taken on a BX51 microscope (Olympus) with UplanFl 60× NA 1.25 oil immersion objective and CCD camera (DP70) or a Nikon TE2000-E with Plan Apo 60× NA 1.4 oil immersion objective and CCD camera (Hamamatsu Coolsnap ES2). Image analysis was carried out using Image J and the data were quantified with Excel (Microsoft) or Origin 7.0 (OriginLab). P values were calculated using a two-tailed, unpaired T-test.

### Plasmids, Chemicals, Antibodies

GFP-tagged versions of the dynein dominant negative constructs P50/dynamitin and dynein intermediate chain IC2C (GFP-IC2-N237) were a gift from Dr. T Shroer [54]. Dominant negative constructs of the kinesin-14 family members KIFC1, KIFC2, and KIFC3 were made by subcloning the stalk and tail regions into pRK5 vector [KIFC1(1–310), KIFC2(1–410), KIFC3(1–375)]. The photoactivatable GFP (PAGFP) plasmid was a gift from Dr. J Lipincott-Schwartz, NIH [102] and was fused in frame with KHC by replacing the fluorescent proteins in mCit-KHC and KHC-mCit [63]. The mCit-GST-NUS plasmid was created by cloning glutathione-S-transferase from pGEX-5X (GE Healthcare) and Nus from pET-44 (Novagen) into the mCit-C1 vector [63] with short linkers between PAGFP and GST (SGGGAA) and GST and NUS (GGAALQ).

Ciliobrevin A (HBI-4) was purchased from Sigma (#H4541, 30 µM final concentration). Lactacystin was purchased from Cayman Chemical (#70980, 5 µM final concentration). All other inhibitors were purchased from Calbiochem: ALLN (N-Acetyl-Leu-Leu-Nle-CHO, 2.3 µM final), Calpain inhibitor III (5.5 µM final), Calpeptin (2 µM final), EST [(2S,3S)-trans-Epoxysuccinyl-L-leucylamido-3-methylbutane Ethyl Ester Loxistatin, 12 µM final], PD150606 (0.5 µM final), and MG132 (20 µM final). The following antibodies were used: β-tubulin (E7, Developmental Studies Hybridoma Bank), giantin (Covance), β-catenin (Sigma), FEZ1 [51], ubiquitin (Zymed), KHC (MAb1614, Chemicon). Secondary antibodies for immunofluorescence were purchased from Jackson ImmunoResearch.

### Live-cell Imaging

CAD cells were plated onto poly-D-lysine-coated glass-bottomed dishes (MatTek) in the absence of serum to differentiate, transfected with TransIT-LTI (Mirus), and imaged 48 hours post-transfection on an Olympus FV-500 confocal microscope with a planapochromat X63/NA 1.4 oil-immersion objective lens. The stage was equipped with a live-cell chamber to maintain 37°C and 5% CO_2_.

For imaging of KHC-PAGFP, three pre-activation GFP images were taken at 4% Argon laser power, 488 nm excitation, with a BA505–525 nm emission filter. Photoactivation of PAGFP in a Region Of Interest (ROI) was performed with a 405 nm laser diode at 100%. For one time photoactivation, GFP images for the entire field of view were then collected every minute under pre-activation conditions. For multiple photoactivation events, GFP images were collected for the entire field of view every minute for four minutes. Then the ROI photoactivation and entire-field GFP imaging steps were repeated. At the completion of GFP image acquisition, the entire field of view was photoactivated with 405 nm laser diode at 100% to visualize the distribution and expression level of KHC-PAGFP in that cell. Cells were included in the data analysis only if they had a normal morphology with neurites whose length was equal to or greater than the diameter of the cell body, and where the KHC-PAGFP protein was expressed at low levels and with a subcellular distribution similar to the endogenous kinesin-1 protein. Images were processed with ImageJ (NIH) and quantified with Excel (Microsoft) and Origin 7.0 (OriginLab).

For FRAP, CAD cells were split at 1.5×10^5^ into glass bottom dishes into Differentiation media and transfected with 2 µg DNA (mCit-KHC, KHC-mCit, or mCit-GST-NUS) 3 h later. After three days, the cells were imaged on an Olympus Fluoview 500 confocal microscope with an Argon laser and DM405-440/515 filter. Only cells with low levels of protein expression were imaged. The entire cell was imaged three times with 10% laser intensity. Then, a 20-30 µm region of the neurite was bleached by scanning 10 times at 100% laser intensity. Fluorescence recovery was measured by imaging the entire cell for 90 seconds at three second intervals with 10% laser intensity. The fluorescence of the bleached area was measured and corrected for photobleaching based on the decrease in fluorescence in a nearby non-beached area of the neurite. To determine the diffusion coefficients of mCit-GST-Nus and mCit-KHC, the recovery kinetics were fit by an analytical solution.

### Computational Modeling

Computer simulations were done using Virtual Cell [103].

## Supporting Information

Figure S1 **Kinesin-1 is not rapidly degraded.** (A,B) Kinesin-1 is not degraded by the proteasome in primary neurons. Primary cortical neurons (7 DIV) were treated with the proteasome inhibitor MG132 for the indicated times. (A) Total cell lysates were separated by SDS-PAGE and blotted with antibodies to ubiquitin, β-catenin, KHC, and tubulin. (B) The average protein levels of KHC (black bars), ubiquitin (light gray bars) and β-catenin (dark gray bars) were quantified from 4 experiments. Error bars indicate SSEM. **, p<0.01 and *, p<0.05 as compared to 0 hr. Although MG132 treatment blocked proteasome activity (increase in ubiquitinated proteins and β-catenin levels over time), the levels of KHC were not significantly affected. (C–E) Kinesin-1 is not degraded by calpains. Differentiated CAD cells were treated with a panel of calpain inhibitors (ALLN, Calpain inhibitor III, Calpeptin, EST, and PD150606) for 16 hrs. (C) Soluble protein lysates were separated by SDS-PAGE and immunoblotted with a monoclonal antibody (H2) to KHC. Immunoblotting with an antibody to α-tubulin was used as a control. (D) Representative images of differentiated CAD cells untreated or treated with PD150606 for 16 hrs and stained with a monoclonal antibody to KHC. The average level of KHC fluorescence in neurite tips was quantified and is presented in (E). *n* = 15 neurites each. Error bars indicate SEM.
(TIF)

Figure S2 **The subcellular distribution of kinesin-1 is not altered upon expression of dominant interfering forms of minus end-directed motors.** (A,B) Differentiated CAD cells were transfected with a plasmid encoding a dominant negative (DN) form of cytoplasmic dynein (GFP-IC2-N237). (A) After 48 hrs, the cells were fixed and stained with a polyclonal antibody to the Golgi marker giantin. Yellow asterisks indicate the nuclei of transfected cells. Dotted pink lines indicate the nuclei of untransfected control cells. Quantification of the data is provided on the far right panel where cells were scored as having a normal or displaced Golgi morphology and then the GFP-IC2-N237 fluorescence was measured. Normal Golgi morphology, *n* = 69 cells, avg. GFP-IC2-N237 fluorescence 375.06±25.26 (SEM). Displaced Golgi morphology, *n* = 38 cells, avg GFP-IC2-N237 fluorescence 2323.17±193.85 (SEM). (B) After 48 hrs, the cells were fixed and stained with a monoclonal antibody to KHC (H2). Yellow arrows, neurite tips of transfected cells; pink arrowheads, neurite tips of untransfected cells. Scale bars, 20 µm. (C) Differentiated CAD cells were transfected with plasmids encoding Myc-tagged DN forms of the kinesin-14 motors KIFC1 [Myc-KIFC1(1–310)], KIFC2 [Myc-KIFC2(1–410)], or KIFC3 [Myc-KIFC3(1–375)]. After 48 hrs, the cells were fixed and co-stained with antibodies to KHC and the Myc tag. Yellow arrows, neurite tips of transfected cells; pink arrowheads, neurite tips of untransfected cells. Scale bar, 20 µm. (D) Quantification of the average fluorescence intensity of KHC in neurite tips in cells expressing DN dynein or kinesin-14 motors. Data are presented as the average KHC fluorescence relative to untransfected cells (set at 100%). Dynein DN, *n* = 15 cells, p = 0.846 compared to untransfected cells; KIFC1 DN, n = 49 cells, p = 0.254 compared to untransfected cells; KIFC2 DN, *n* = 60 cells, p = 0.001 compared to untransfected cells; KIFC3 DN, *n* = 49 cells, p = 0.343 compared to untransfected cells (Student's t-test). The fact that expression of DN KIFC2 caused a decrease in the average fluorescence intensity of kinesin-1 indicates that KIFC2 could impact anterograde transport but does not play a role in recycling of kinesin-1 motors. The fact that knockout of the KIFC2 gene in mice has no phenotype [104], is consistent with this conclusion.
(TIF)

Figure S3 **Levels of FP-KHC expression as compared to endogenous levels.** To ensure that the behavior of expressed KHC-mCit reflects that of the endogenous kinesin-1 motor, we imaged cells with low levels of FP-KHC expression. To do this, we first analyzed FP-KHC expression in fixed cells. Differentiated CAD cells were transfected with plasmids for KHC-mCit and then 48 hrs later, the cells were fixed and stained with a monoclonal antibody that recognizes KHC from all three genes (KIF5A, KIF5B, and KIF5C). Wide-field microscopy images were taken with identical exposure conditions. Yellow arrows, neurite tips of transfected cells; pink arrowheads, neurite tips of untransfected cells. In cells expressing low levels of KHC-mCit (top panels), the expressed protein was primarily localized in the neurite tips, similar to the endogenous protein distribution. In cells expressing medium levels of KHC-mCit (middle panels), the expressed proteins localize to the cell bodies and in cells with high levels of expression (bottom panels), KHC-mCit localized throughout the cell body, neurite shaft and and neurite tip. Thereafter, cells expressing low levels of KHC-mCit were selected for analysis based on the localization of the expressed protein primarily to the neurite tips.
(TIF)

Figure S4 **Determination of the kinesin-1 diffusion coefficient.**
(PDF)

Figure S5 **Modeling of kinesin-1 turnover in neuronal processes.**
(PDF)
