# Supplementary figures and images for: Recycling of Kinesin-1 Motors by Diffusion after Transport
Source: PLoS One. 2013 Sep 30;8(9):e76081. doi: 10.1371/journal.pone.0076081 (PMC3786890; doi:10.1371/journal.pone.0076081)

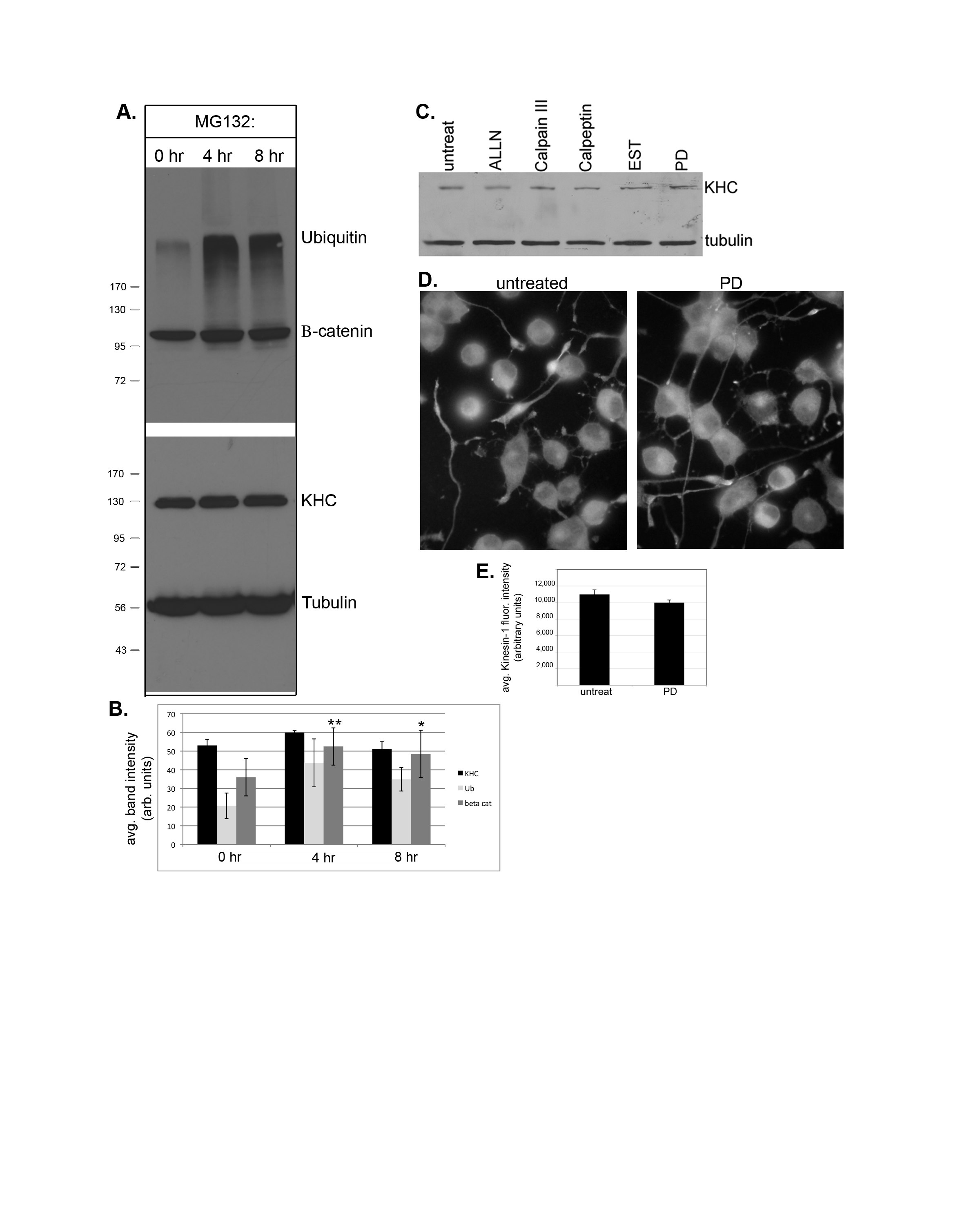

Supplement: Figure S1 — Kinesin-1 is not rapidly degraded. (A,B) Kinesin-1 is not degraded by the proteasome in primary neurons. Primary cortical neurons (7 DIV) were treated with the proteasome inhibitor MG132 for the indicated times. (A) Total cell lysates were separated by SDS-PAGE and blotted with antibodies to ubiquitin, β-catenin, KHC, and tubulin. (B) The average protein levels of KHC (black bars), ubiquitin (light gray bars) and β-catenin (dark gray bars) were quantified from 4 experiments. Error bars indicate SSEM. **, p<0.01 and *, p<0.05 as compared to 0 hr. Although MG132 treatment blocked proteasome activity (increase in ubiquitinated proteins and β-catenin levels over time), the levels of KHC were not significantly affected. (C–E) Kinesin-1 is not degraded by calpains. Differentiated CAD cells were treated with a panel of calpain inhibitors (ALLN, Calpain inhibitor III, Calpeptin, EST, and PD150606) for 16 hrs. (C) Soluble protein lysates were separated by SDS-PAGE and immunoblotted with a monoclonal antibody (H2) to KHC. Immunoblotting with an antibody to α-tubulin was used as a control. (D) Representative images of differentiated CAD cells untreated or treated with PD150606 for 16 hrs and stained with a monoclonal antibody to KHC. The average level of KHC fluorescence in neurite tips was quantified and is presented in (E). n = 15 neurites each. Error bars indicate SEM. (TIF) [file pone.0076081.s001.tif]

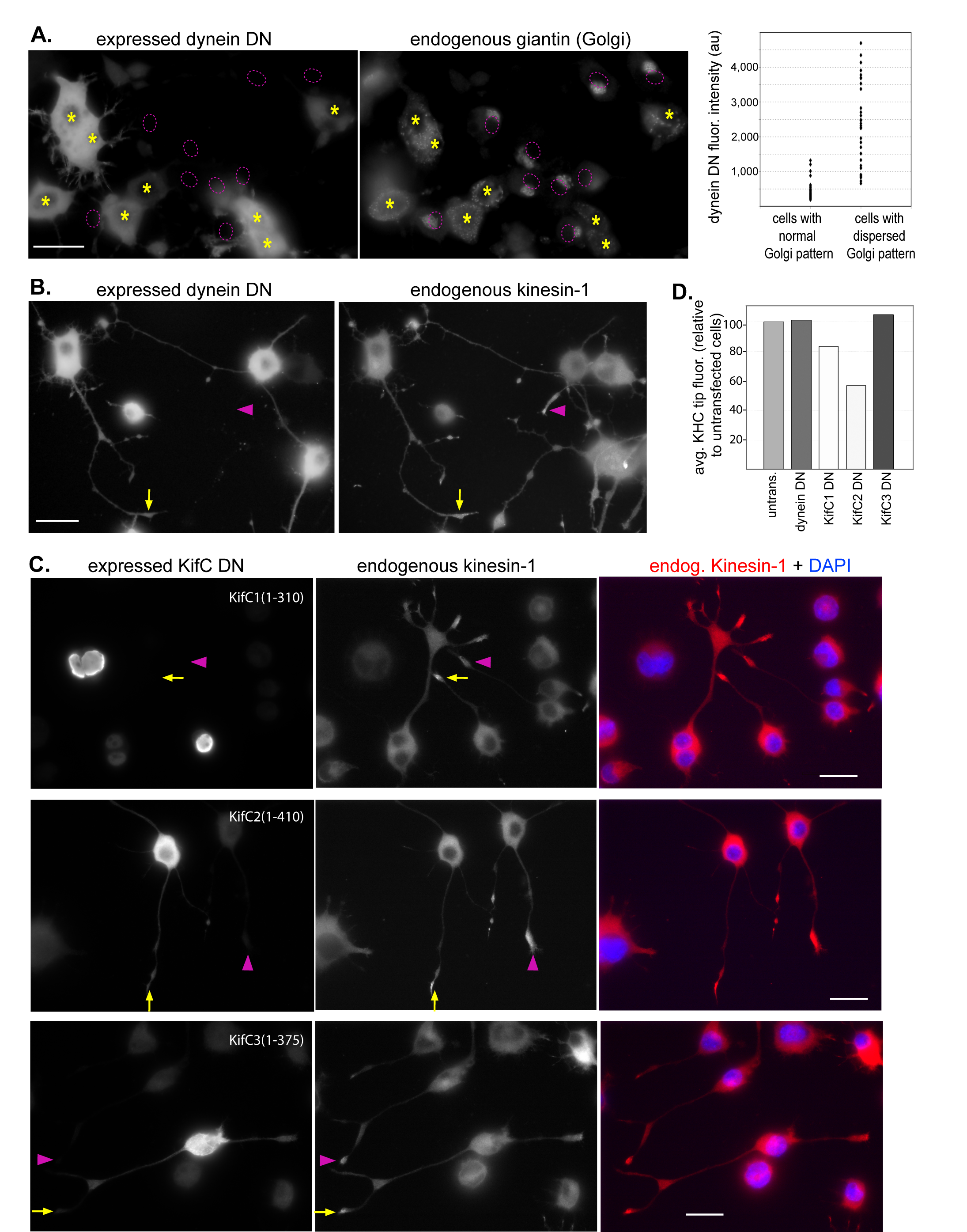

Supplement: Figure S2 — The subcellular distribution of kinesin-1 is not altered upon expression of dominant interfering forms of minus end-directed motors. (A,B) Differentiated CAD cells were transfected with a plasmid encoding a dominant negative (DN) form of cytoplasmic dynein (GFP-IC2-N237). (A) After 48 hrs, the cells were fixed and stained with a polyclonal antibody to the Golgi marker giantin. Yellow asterisks indicate the nuclei of transfected cells. Dotted pink lines indicate the nuclei of untransfected control cells. Quantification of the data is provided on the far right panel where cells were scored as having a normal or displaced Golgi morphology and then the GFP-IC2-N237 fluorescence was measured. Normal Golgi morphology, n = 69 cells, avg. GFP-IC2-N237 fluorescence 375.06±25.26 (SEM). Displaced Golgi morphology, n = 38 cells, avg GFP-IC2-N237 fluorescence 2323.17±193.85 (SEM). (B) After 48 hrs, the cells were fixed and stained with a monoclonal antibody to KHC (H2). Yellow arrows, neurite tips of transfected cells; pink arrowheads, neurite tips of untransfected cells. Scale bars, 20 µm. (C) Differentiated CAD cells were transfected with plasmids encoding Myc-tagged DN forms of the kinesin-14 motors KIFC1 [Myc-KIFC1(1–310)], KIFC2 [Myc-KIFC2(1–410)], or KIFC3 [Myc-KIFC3(1–375)]. After 48 hrs, the cells were fixed and co-stained with antibodies to KHC and the Myc tag. Yellow arrows, neurite tips of transfected cells; pink arrowheads, neurite tips of untransfected cells. Scale bar, 20 µm. (D) Quantification of the average fluorescence intensity of KHC in neurite tips in cells expressing DN dynein or kinesin-14 motors. Data are presented as the average KHC fluorescence relative to untransfected cells (set at 100%). Dynein DN, n = 15 cells, p = 0.846 compared to untransfected cells; KIFC1 DN, n = 49 cells, p = 0.254 compared to untransfected cells; KIFC2 DN, n = 60 cells, p = 0.001 compared to untransfected cells; KIFC3 DN, n = 49 cells, p = 0.343 compared to untransfected cells [file pone.0076081.s002.tif]

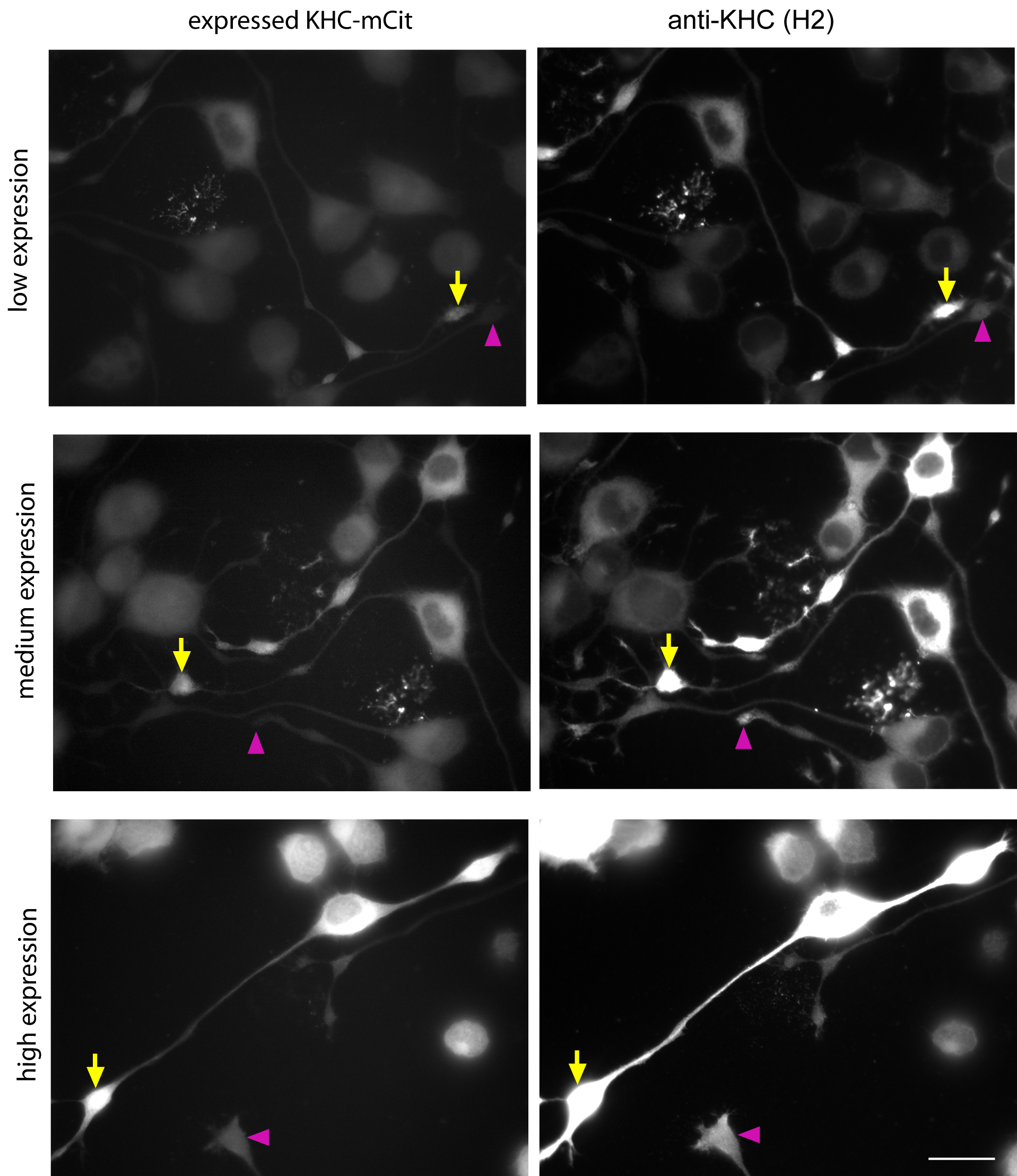

Supplement: Figure S3 — Levels of FP-KHC expression as compared to endogenous levels. To ensure that the behavior of expressed KHC-mCit reflects that of the endogenous kinesin-1 motor, we imaged cells with low levels of FP-KHC expression. To do this, we first analyzed FP-KHC expression in fixed cells. Differentiated CAD cells were transfected with plasmids for KHC-mCit and then 48 hrs later, the cells were fixed and stained with a monoclonal antibody that recognizes KHC from all three genes (KIF5A, KIF5B, and KIF5C). Wide-field microscopy images were taken with identical exposure conditions. Yellow arrows, neurite tips of transfected cells; pink arrowheads, neurite tips of untransfected cells. In cells expressing low levels of KHC-mCit (top panels), the expressed protein was primarily localized in the neurite tips, similar to the endogenous protein distribution. In cells expressing medium levels of KHC-mCit (middle panels), the expressed proteins localize to the cell bodies and in cells with high levels of expression (bottom panels), KHC-mCit localized throughout the cell body, neurite shaft and and neurite tip. Thereafter, cells expressing low levels of KHC-mCit were selected for analysis based on the localization of the expressed protein primarily to the neurite tips. (TIF) [file pone.0076081.s003.tif]
