# Supplementary material for: Recycling of Kinesin-1 Motors by Diffusion after Transport
Source: PLoS One. 2013 Sep 30;8(9):e76081. doi: 10.1371/journal.pone.0076081 (PMC3786890; doi:10.1371/journal.pone.0076081)
Supplement: Figure S4 — Determination of the kinesin-1 diffusion coefficient. (PDF) [file pone.0076081.s004.pdf]

Figure S4. Determination of the kinesin-1 diffusion coefficient.

Fluorescence recovery after photobleaching (FRAP) was carried out for differentiated CAD cells expressing low levels of KHC-mCit. The diffusion coefficient of KHC was then determined in two ways:

1) Fluorescence recovery data for each cell were fit to an analytical solution over the entire recovery time (30 s) (Fitting worksheet.xls).

The exact solution for the recovery after bleaching a segment of length 2*a* of an infinite one-dimensional axon is:

$$F(t) = F(0) + (F_\infty - F(0))g(a/\sqrt{Dt}),$$

where $F(t)$ is the fluorescence averaged over the bleached region at time *t*, F_∞ is the final recovery level, $F(0)$ is the fluorescence averaged over the bleached region at the first post-bleach time point , and

$$g(x) = 1 - erf(x) + (1 - e^{-x^2})/(x\sqrt{\pi}) \quad \text{with} \quad erf(x) = (2/\sqrt{\pi})\int_0^x e^{-t^2}\,dt\,.$$

The figure below shows the analytical recovery curve when the conditions are set at 2*a* =24 μm, *D* = 5 μm^2/sec, and $F(0) = 0$.

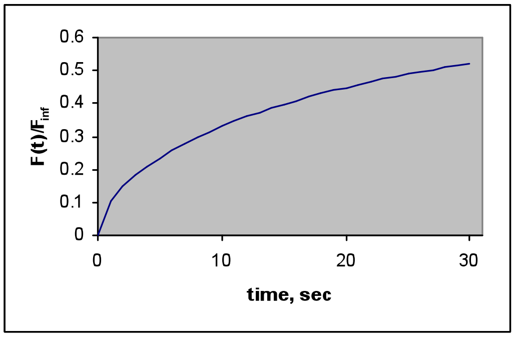

Fitting of the experimental data was performed by varying two parameters: the diffusion coefficient and the recovery level (mobile fraction) (Analysis_20090428_FRAPKHCmCit.xls). Prior to fitting, the data was corrected for fluorescence loss due to bleaching while collecting images. This was done by monitoring the decrease in fluorescence of a nearby unbleached area. The example, below, illustrates the quality of fit for one cell.

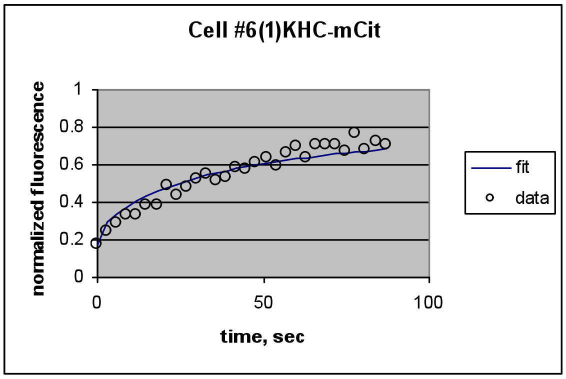

Averaging the results from eight cells yielded the diffusion coefficient of 6.4 ± 0.9 $\mu m^2/s$. The average fitting error (the mean absolute difference between the data and the fitting curve) was relatively large: 0.035, likely due to the participation of a fraction of the KHC-mCit motors in active transport.

2) Fluorescence recovery data for each cell were fit to an analytical solution over the first 15 sec of recovery (Fitting worksheet.xls).

Because diffusion is faster than directed transport on small spatial and temporal scales, the contribution of active transport to the fluorescent recovery can be minimized by applying the fitting procedure to the first 15 seconds of the recovery. The choice of the time cutoff reflects the trade-off between the effects of active transport (significant at longer time scales) and measurement error (significant at shorter time scales). Fitting the KHC-mCit recovery data to an analytical solution over the first 15 sec yielded the value 4.08 ± 0.46 $\mu m^2/s$ for the diffusion coefficient of kinesin, with the average error of fit of 0.016. This number is closer to 4.11 ± 0.54 $\mu m^2/s$ obtained for the diffusion coefficient of mCit-GST-NUS, a construct similar to KHC except that it does not bind to cargoes or microtubules. The diffusion coefficient for mCit-GST-NUS was obtained by applying the same fitting procedure to the recovery data over the entire measurement time (30s). We therefore believe that 4.08 ± 0.46 $\mu m^2/s$ is an accurate estimate of the diffusion coefficient of KHC in the process of a differentiated CAD cell.
