# Supplementary material for: Recycling of Kinesin-1 Motors by Diffusion after Transport
Source: PLoS One. 2013 Sep 30;8(9):e76081. doi: 10.1371/journal.pone.0076081 (PMC3786890; doi:10.1371/journal.pone.0076081)
Supplement: Figure S5 — Modeling of kinesin-1 turnover in neuronal processes. (PDF) [file pone.0076081.s005.pdf]

Figure S5. Modeling of kinesin-1 turnover in neuronal processes

Part 1. The Diligent Worker and Loose Bucket Brigade models

1A. Diligent Workers

This model assumes that cargo-bound kinesin-1 motors bind to a microtubule at its minus end in the cell body and move actively all the way to the plus end of the microtubule in the axon terminal where they unbind from both cargo and microtubule and diffuse back. That is, anterograde motors move only by active transport at v= 0.78 μm/s [1] whereas retrograde motors move only by diffusion at D=4.08 ± 0.46 μm^2/s (Figure S4). Binding of kinesin-1 motors (k_{on}) to the microtubule track occurs only at the minus ends in the cell body whereas unbinding from the track (k_{off}) occurs only at the plus ends in the axon terminal.

Let U and B denote the local fractions of unbound and bound motors, respectively. Their steady-state distributions along a neurite are described by the following equations:

$$\partial_{xx}U = 0,\ \partial_x B = 0$$

The solutions are:

$$U(x) = U_- + (U_+ - U_-)x/L,\ \ x \in [0, L],\ \text{and}\ B = const \equiv B_0,$$

where U_- and U_+ are the fractions of the unbound motors at the minus and plus ends, respectively, and L is the axon length. Thus, using the equation

$$F_{\text{tot}}(x) \equiv U(x) + B_0 = U_- + B_0 + (U_+ - U_-)x/L. \qquad \mathbf{(1)}$$

demonstrates that the Diligent Worker mechanism results in a linear distribution of the total kinesin-1 density along the axon (Figure S5A, left panel).

The constant values U_-, U_+, and B_0 in Eq (1) are determined from boundary conditions based on the following assumptions. First, the diffusive flux of kinesin-1, $-D\partial_x U$, must balance the active flow, vB (D and v are the diffusion coefficient and the average velocity, respectively), yielding

$$U_+ - U_- = \frac{vL}{D}B_0.$$

Second, the fraction of kinesin-1 motors bound to cargo must be constant in time. That is, the rate of binding at the minus end, $k_{\text{on}}U_-$, should balance the rate of unbinding at the plus end,

$$k_{\text{off}}B_0 : \ U_- = \frac{k_{\text{off}}}{k_{\text{on}}}B_0.$$

Finally, the total number of kinesin-1 motors in the neurite must be conserved, $\int_0^L F_{\text{tot}}(x)dx = \overline{F}L$,

resulting in $B_0 = \dfrac{\overline{F}}{1 + k_{\text{off}}/k_{\text{on}} + vL/2D}$.

Eq **(1)** then takes the form: $F_{\text{tot}}(x) = \overline{F}\dfrac{1 + k_{\text{off}}/k_{\text{on}} + vx/D}{1 + k_{\text{off}}/k_{\text{on}} + vL/2D}$.

The recycling of kinesin-1 motors is described by two characteristics, the steady-state turnover rate R and the time per kinesin-1 cycle τ.

$$R = vB_0 = \frac{v\overline{F}}{1 + k_{\text{off}} / k_{\text{on}} + vL / 2D},$$

$$\tau = \frac{\overline{F}L}{vB_0} \sim \frac{L}{v} + \frac{L^2}{2D}.$$

Whereas Diligent Workers are effective in targeting cargos produced in the cell body to the neurite tips, their turnover rate R slows down for longer axons. For axonal lengths greater than $2D/v \sim 10$ μm, diffusion becomes a rate limiting factor: $\tau \sim L^2/2D$. Still, for axonal lengths ~ 60-70 μm, the time of recycling is only ~10 min which is within the time frame of kinesin-1-1dependent transport events [2]. However, in processes longer than ~ 100 μm, diffusion of kinesin-1 motors within the Diligent Worker model limits the rate of kinesin-1 recycling.

1B. Loose Bucket Brigade

This model assumes that both anterograde and retrograde motors participate in active transport and diffusion. Binding/unbinding of kinesin-1 motors to/from the microtubule track occurs throughout the axon. In this scenario, the cargo frequently “changes hands”, as the driving motor spontaneously unbinds from the cargo and microtubule, undergoes a period of diffusive movement, and then reattaches to a cargo and reactivates. Thus, this model is termed a “bucket brigade”.

As in the Diligent Worker model, the motor bound to a cargo actively moves along a microtubule in the plus end direction with velocity v= 0.78 μm/s. Upon random unbinding characterized by frequency k_{off}, the motor undergoes free diffusion in the cytoplasm with the diffusion coefficient D=4.08 ± 0.46 μm^2/s. The diffusive motion, in turn, is randomly interrupted by binding events. We assume that the frequency k_{on} of binding events is constant throughout the axon, i.e. cargo is in constant and uniform supply along the axon.

In this model, which is equivalent to a two-state model earlier used to analyze kinesin distributions in *in vitro* microtubule assays [3], the steady state of the system is described by the following differential equations:

$$\begin{aligned} &D\partial_{xx}U - k_{\text{on}}U + k_{\text{off}}B = 0, \\ &-v\,\partial_x B + k_{\text{on}}U - k_{\text{off}}B = 0, \end{aligned} \qquad \textbf{(2)}$$

with the notation as above.

To solve Eqs (2), we first sum up Eqs (2) and take into account that the net flux of the motors is zero. This yields

$$B = (D/v)\partial_x U, \qquad \textbf{(3)}$$

and the problem reduces to determining $U(x)$. The equation for $U(x)$ is obtained by substituting Eq (3) back into the first of Eqs (2):

$$\partial_{xx}U + (k_{\text{off}}/v)\partial_x U - (k_{\text{on}}/D)U = 0.$$

The general solution of this equation is

$$U(x) = c_1 \exp(x/\lambda_1) + c_2 \exp(-x/\lambda_2), \qquad \textbf{(4)}$$

where $\lambda_1 = s(\sqrt{(s/2l)^2+1} + s/2l)$ and $\lambda_2 = s(\sqrt{(s/2l)^2+1} - s/2l)$ are the solutions of the characteristic equation $\lambda^{-2} + l^{-1}\lambda^{-1} - s^{-2} = 0$.

Here , $s = \sqrt{D/k_{\text{on}}}$ is a "diffusion length" between the consecutive events of unbinding and binding and $l = v/k_{\text{off}}$ is the average run length of the motor on MT.

Eq (4) is dominated by the first term, $U(x) \approx U(0)\exp(x/\lambda_1)$. Then with the account of Eq (3), the total concentration of active and inactive kinesin is

$$F_{\text{tot}}(x) \equiv U(x) + B(x) \approx F_0 \exp(x/\lambda_1),$$

where the pre-factor F_0 $(= F_{\text{tot}}(0))$ can be expressed in terms of the average concentration $\overline{F}$ of total kinesin (active and inactive) in the system. Indeed, given that the total number of kinesins is conserved, $\int_0^L F_{\text{tot}}(x)dx = \overline{F}L$, $F_0 \approx \overline{F}\frac{L}{\lambda_1}(e^{L/\lambda_1} - 1)^{-1}$.

Overall, the steady-state spatial profile of total kinesin for the "bucket brigade" with constant frequency of activation is described by the following equation,

$$F_{\text{tot}}(x) = \frac{\overline{F}L\exp(x/\lambda_1)}{\lambda_1(\exp(L/\lambda_1) - 1)}. \qquad \textbf{(5)}$$

Using Eq (5) we find that in agreement with [3], the Loose Bucket Brigade mechanism yields an exponential steady-state profile of kinesin-1 motor distribution along the axon (Figure S5A, right panel).

The corresponding turnover rate, $R = vB(L) = \frac{D}{(\lambda_1 + D/v)} \cdot \frac{\overline{F}L}{\lambda_1(1 - \exp(-L/\lambda_1))}$, increases with axonal length, and the time per cycle becomes essentially independent of L for long axons:

$$\tau = \frac{\overline{F}L}{R} \approx \frac{\lambda_1}{v} + \frac{\lambda_1^{\,2}}{D} \text{ for } L >> \lambda_1.$$

While the turnover parameters of the bucket brigade are better than those of the diligent workers, the bucket brigade loses to diligent workers in targeting cargos in the cell body. Specifically, the fraction of the total flux made up by the cargos from the cell body, $\frac{B(0)}{B(L)} = e^{-L/\lambda_1}$, becomes exponentially small for long axons. For $L = 10\lambda_1$, for example, this fraction is only 4.5x10^{-3} %. This is due to the sloppiness of the bucket brigade: upon dissociation from the cargo and MT, the motor does not necessarily return to where it was activated but rather can diffuse in either direction.

The spatial profile in the bucket brigade scenario, Eq (5), is largely defined by the parameter λ_1 which depends both on the association/dissociation rate constants k_{on} and k_{off} and the transport parameters D and v (see the equations that follow Eq (4)). For $\lambda_1 < L$, the distribution profiles along an axon are very different for kinesin-1 motors as diligent workers versus in bucket brigade (Figure S5A) with the same set of parameters: v = 0.78 μm/s, D = 4.08 μm^2/s, k_{on} =

1.22 s^{-1}, k_{off} = 2/3 s^{-1}, and $\lambda_1 / L \approx 0.1$. However, if $\lambda_1 >> L$, the profiles in the two scenarios become similar.

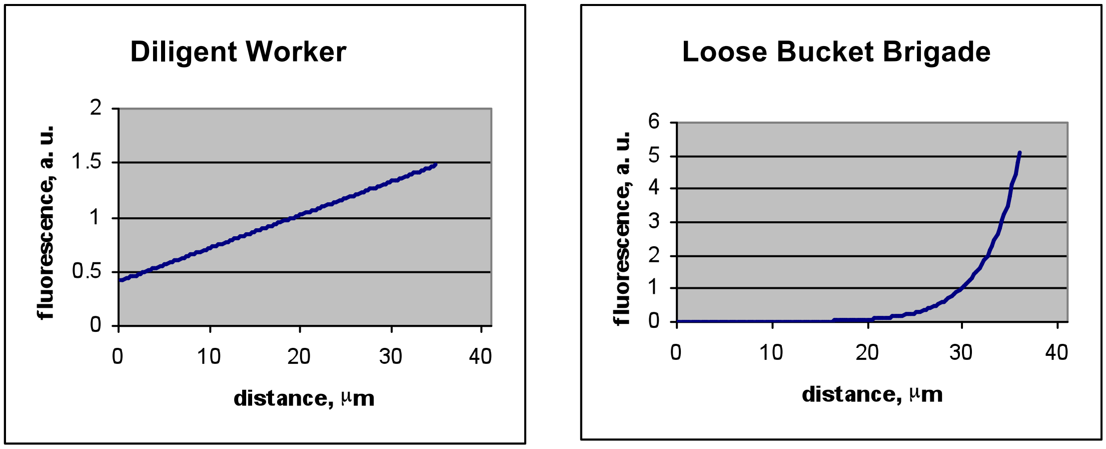

Figure S5A. Predicated distribution of the total fraction of kinesin-1 motors along an axon in the Diligent Worker scenario (left panel) versus Loose Bucket Brigade scenario (right panel).

Part 2. Experimental determination of the KHC spatial distribution is consistent with a Loose Bucket Brigade mechanism of kinesin recycling.

To determine the spatial distribution of the total kinesin-1 in neuronal cell processes, CAD cells and primary hippocampal neurons were stained with a monoclonal antibody (H2) that recognizes a sequence in the stalk domain of all three KHC-encoding gene products (KIF5A, KIF5B, KIF5C) regardless of their conformation (autoinhibited versus active) ([4] and data not shown). CAD cells were chosen as a "simplified" neuron as they generate one or more neurites that appear equal in their kinesin-1-dependent transport. Primary hippocampal neurons were used to verify the CAD cell results in true axons. Primary hippocampal neurons grown for 2 days in vitro (2DIV) generate a single axon and several minor neurites whereas cells grown for 9DIV contain a single axon and several dendrites that have begun to synapse with axons of adjacent cells [5].

To correct for any changes in KHC fluorescence signal intensity due to changes in cell volume, CAD cells and primary hippocampal neurons were first transfected with plasmids expressing enhanced green fluorescent protein (EGFP). The cells were fixed and stained with the H2 antibody and the amount of KHC protein was quantified as a ratio of the raw KHC fluorescence intensity and the corresponding EGFP fluorescence intensity for each pixel along the neurite (CAD cells) or axon (primary hippocampal neurons). Figure S5B shows the increasing portion of the curve at the end of the neurite of one CAD cell. A typical qualitative feature of the KHC distribution in this and all other cells is an exponential increase of concentration towards the microtubule plus end in the axon terminal. Thus, the experimentally-observed kinesin spatial profiles appear to point in favor of the Loose Bucket Brigade.

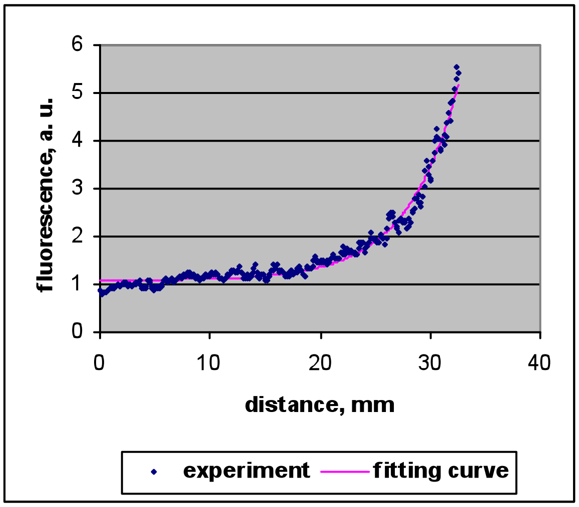

Figure S5B. Experimentally-determined distribution of kinesin-1 motors in the neurite process of a differentiated CAD cell. Pink line, exponential fit of the data using the formula $F(x) = F_0 \exp(x / \lambda_1) + F_1$. **The average relative error of fit is < 9%.**

One of the fitting parameters is the characteristic length λ_1. The value of λ_1 averaged over 12 CAD cells is 3.76 μm. Based on the values of λ_1 , the diffusion coefficient D (≈ 4.08 μm^2/s, Figure S4), the kinesin velocity (0.78 μm/s, [1]) and the average run length l (≈ 1.17 μm [1]) allows one to estimate k_{on} (see the equations following Eq (4)): $k_{on} = \frac{D}{\lambda_1}(\frac{1}{\lambda_1} + \frac{1}{l}) \approx 1.22\ s^{-1}$.

The experimentally-observed exponential increase in KHC protein at axon tips matches the predicted distribution of kinesin-1 in the Loose Bucket Brigade but not the Diligent Worker model (Figure 5). However, in neurites and axons, kinesin-1 also displays an increase at the minus ends of the microtubules (at the cell body, Figure 5E) that is not predicted in either of the models. The experimentally-observed increase in kinesin-1 protein at the microtubule minus ends could be due to motors that do not participate in active transport or could simply be due to background fluorescence. To distinguish between these possibilities, control experiments were performed by omitting the H2 primary antibody from the staining protocol. The residual fluorescence at the minus ends of the microtubules in the absence of anti-kinesin-1 antibodies (Figure S5C) indicates that the baseline component is essentially due to background fluorescence. Thus, all kinesin-1 motors appear to participate in both active transport and free diffusion.

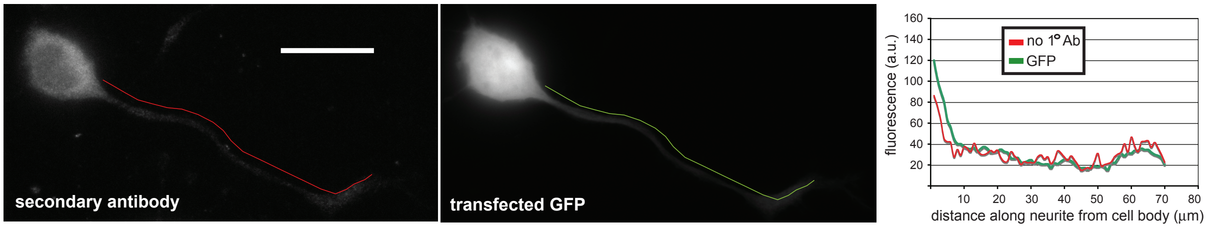

Figure S5C. Differentiated CAD cells expressing EGFP (a marker of cell volume) were immunostained as in Figure 5 except that the H2 primary antibody was omitted. The fluorescence intensities due to the secondary antibody (red line) and EGFP (green line) were plotted along the length of the neurite (right panel). Scale bar, 20 μm.

Part 3. Hypothesis of an "organized bucket brigade"

Both the Diligent Worker and the Loose Bucket Brigade models have their strengths and weaknesses. Diligent Workers bind 100% of cargoes generated in the cell body but their turnover rate in long axons, dominated by diffusion, would be prohibitively slow. In contrast, in the Loose Bucket Brigade, kinesin-1 recycling is independent of the axon length but for long axons, it is ineffective with respect to targeting cargoes in the cell body (still, for $L = 4\lambda_1$, the fraction of flux due to cargoes generated in the cell body is $\approx$ 2 %).

One way to overcome the slowness of diffusion would be to "organize" the bucket brigade by assigning kinesin-1 molecules to a particular segment of the axon. For this, the diffusion of inactive kinesin-1 motors should be "rectified", i.e. the dissociated kinesin-1 motors must not diffuse in the "wrong" direction. The idea of diffusion rectification is not unheard of in applications to cellular systems, e. g. Brownian ratchet [6]. To preclude kinesin-1 from diffusing in the plus end direction, there should be diffusion barriers of some sort. These could be pile-ups of large cargoes at the ends of microtubule fragments or, probably more likely, stochastic cargo accumulations [7] sufficient for obstructing diffusion. Our preliminary models of organized bucket brigades indicate that this mechanism indeed overcomes deficiencies of a loose bucket brigade and presents a diffusion-based alternative to models of active recycling of kinesin.

References

1. Cai D, Verhey KJ, Meyhofer E (2007) Tracking single Kinesin molecules in the cytoplasm of mammalian cells. Biophys J 92: 4137-4144.
2. Reed NA, Cai D, Blasius TL, Jih GT, Meyhofer E, et al. (2006) Microtubule acetylation promotes kinesin-1 binding and transport. Curr Biol 16: 2166-2172.
3. Nedelec F, Surrey T, Maggs AC (2001) Dynamic concentration of motors in microtubule arrays. Phys Rev Lett 86: 3192-3195.
4. DeBoer SR, You Y, Szodorai A, Kaminska A, Pigino G, et al. (2008) Conventional kinesin holoenzymes are composed of heavy and light chain homodimers. Biochemistry 47: 4535-4543.
5. Dotti CG, Sullivan CA, Banker GA (1988) The establishment of polarity by hippocampal neurons in culture. J Neurosci 8: 1454-1468.
6. Peskin CS, Odell GM, Oster GF (1993) Cellular motions and thermal fluctuations: the Brownian ratchet. Biophys J 65: 316-324.
7. Leduc C, Padberg-Gehle K, Varga V, Helbing D, Diez S, et al. (2012) Molecular crowding creates traffic jams of kinesin motors on microtubules. Proc Natl Acad Sci U S A 109: 6100-6105.
